# Why Are Some People with Lower Urinary Tract Symptoms (LUTS) Depressed? New Evidence That Peripheral Inflammation in the Bladder Causes Central Inflammation and Mood Disorders

**DOI:** 10.3390/ijms24032821

**Published:** 2023-02-01

**Authors:** Francis M. Hughes, Michael R. Odom, Anissa Cervantes, Austin J. Livingston, J. Todd Purves

**Affiliations:** Department Urology, Duke University Medical Center, P.O. Box 3831, Durham, NC 27710, USA

**Keywords:** inflammation, lower urinary tract symptoms, bladder, depression, mood disorders, cystitis

## Abstract

Anecdotal evidence has long suggested that patients with lower urinary tract symptoms (LUTS) develop mood disorders, such as depression and anxiety, at a higher rate than the general population and recent prospective studies have confirmed this link. Breakthroughs in our understanding of the diseases underlying LUTS have shown that many have a substantial inflammatory component and great strides have been made recently in our understanding of how this inflammation is triggered. Meanwhile, studies on mood disorders have found that many are associated with central neuroinflammation, most notably in the hippocampus. Excitingly, work on other diseases characterized by peripheral inflammation has shown that they can trigger central neuroinflammation and mood disorders. In this review, we discuss the current evidence tying LUTS to mood disorders, its possible bidirectionally, and inflammation as a common mechanism. We also review modern theories of inflammation and depression. Finally, we discuss exciting new animal studies that directly tie two bladder conditions characterized by extensive bladder inflammation (cyclophosphamide-induced hemorrhagic cystitis and bladder outlet obstruction) to neuroinflammation and depression. We conclude with a discussion of possible mechanisms by which peripheral inflammation is translated into central neuroinflammation with the resulting psychiatric concerns.

## 1. Introduction

An association between lower urinary tract symptoms (LUTS) and mood disorders, such as anxiety and depression, has been suspected for decades. In 1964, Hollander, Gordon and Sloan first noted the relationship between urinary obstruction and what was described as psychotic behavior [1]. Decades later, large population-based studies uncovered a general relationship between LUTS and mood disorders which confirmed the anecdotal evidence that was familiar to most clinicians [2]. Subsequent prospective studies have further defined this relationship [3,4,5,6,7,8], and new preclinical data suggest that inflammation is a critical mechanism whereby stressors in the lower urinary tract produce psychiatric sequelae [9,10]. Not only does this suggest a pathway by which benign urological disease can cause psychological problems, it also explains why pro-inflammatory co-morbidities, such as diabetes and autoimmune diseases, are common in patients suffering from both LUTS and depression/anxiety. This review will focus on the relationship between LUTS and mood disorders with special attention to the role of inflammation as the mediator between the two conditions.

## 2. There Is a Strong Clinical Association between LUTS and Depression/Anxiety

Perhaps the most compelling clinical evidence of the association of LUTS and mood disorders comes from the Epidemiology of LUTS (EpiLUTS) study which was a cross-sectional, population-based study conducted via the internet and composed of respondents from the USA, United Kingdom, and Sweden [2]. In total, 30,000 men and women participated. Results indicate that patients with urinary symptoms had significantly higher Hospital Anxiety and Depression Scores (HADS) than patients who did not report urinary symptoms. Accordingly, 35.9% of men with LUTS reported a clinically significant HADS-A (anxiety) score compared to 10.7% of men with no LUTS. Likewise, 53.3% of women with overactive bladder (OAB) reported a clinical HADS-A score compared to only 18% of women without urinary dysfunction. Similar results were seen for depression scores [2]. Additionally, patients with OAB symptoms deemed “bothersome” conveyed worse anxiety scores than OAB patients who did not find their symptoms to be bothersome [11]. In another study, Tzeng et al. [12] followed patients for up to 13 years and found a 41.7% increase in the incidence of psychiatric diseases (dementia, anxiety, depression, sleep disorders, and psychotic disorders) in patients with OAB compared to their age matched controls. Finally, in one of the largest studies so far, a systematic review of over 80,000 patients found GRADE level 2 evidence (moderate certainty) between the co-occurrence of OAB and psychiatric symptoms [13].

Further evidence for the connection between these disorders can be found in smaller studies focusing on specific populations. Wong et al. published a case–control study of elderly Chinese men that showed moderate-to-severe LUTS was significantly associated with clinically relevant depression [14]. Moreover, analysis of the Boston Area Community Health Survey found depressive symptoms were associated with increased rates of LUTS across all gender, race, and ethnic groups [15]. Meanwhile, a systematic review of the literature between 1990 and 2019 consistently found a significant association between clinical anxiety and LUTS in both men and woman [16]. This association was strong enough that Johnson et al. even suggested physicians could use the International Prostate Symptom Score (IPSS) quality of life (QoL) index as a depression screening tool [17]!

## 3. The Correlation between LUTS and Depression/Anxiety Is Bidirectional

It is most interesting that this association between LUTS and depression does not appear to be unidirectional as patients with depression or mood disorders are also more likely to develop LUTS. This potential bidirectionality has been elucidated in newer, prospective studies. Huang et al. [5] used the Taiwan National Health Insurance Research Database to follow two groups of patients for 3 years. In patients with LUTS at the beginning of the study, they found the expected increased risk of developing anxiety and/or depression, but for patients starting with anxiety or depression they also found an increased risk for development of LUTS. Several systematic reviews of the literature have also looked at this relationship. For example, Martin et al. [13] found a strong association of anxiety among people with LUTS and only a slightly lower association of LUTS among people with depression. In addition, Mahjani et al. [16] found a nearly identical odds ratio of anxiety among people with LUTS or LUTS among people with anxiety, regardless of sex. Thus, there is strong clinical evidence tying these two conditions together, irrespective of which comes first.

## 4. Interventional Studies Support This Correlation

Interventional studies have added important evidence that the presentation of LUTS and depression/anxiety are causally related. In several studies, the investigators treated only LUTS but observed improvement in both conditions. For example, Staskin and colleagues [18] followed men with OAB and depression that were treated with oxybutynin for OAB. After 6 months, improvement was seen in both urinary scores and depression scores. In another study with patients that had recurrent urinary tract infections (UTIs), 61.9% had depression at baseline. Patients that were subsequently prophylaxed with a lyophilized extract of E. coli (OM-89) not only saw urinary symptoms significantly improve but total HADS score decreased by almost a third [19]. A similar phenomenon was seen in patients with benign prostatic hyperplasia (BPH). Quek et al. [20] treated patients with BPH medically (alpha-blockade) or surgically (transurethral resection of the prostate) and found improvement in patient’s urinary as well as anxiety/depression scores. Interestingly, the surgical group, which unsurprisingly had superior improvement in LUTS symptoms compared to the medical group, also showed greater improvement in their mood disorders. Thus, the magnitude of effect on the psychological disorder was directly dependent on the magnitude of effect on the urological disorder.

## 5. Interstitial Cystitis/Bladder Pain Syndrome (IC/BPS)

IC/BPS is the urinary disease most commonly linked to severe psychiatric comorbidities and the literature indicates these patients have a 5–50% rate of depression [21,22,23,24]. In addition, in one study 11% of those with IC/BPS reported episodes of suicidal ideation in just the previous two weeks [25]. While the exploration of a bladder-brain axis may be most compelling for this condition, unfortunately IC/BPS patients can experience severe, debilitating and chronic bladder pain. Chronic pain, in and of itself, is well-established to increase the risk of developing depression [26]. Thus, the inclusion of pain in the constellation of symptoms for IC/BPS does add a critical differentiating component compared to other benign urological conditions. In order to simplify this review, we will avoid further discussion of IC/BPS and other chronic pelvic pain syndromes that would unnecessarily complicate our model.

## 6. Inflammation as a Possible Common Mechanism between LUTS and Depression

### 6.1. Inflammation Is a Well-Established Component of Many Benign Urological Disorders That Cause LUTS

Inflammation is well-known to be involved in many urological disorders. This inflammation can be systemic. For example, patients with systemic inflammation due to obesity, diabetes or autoimmune diseases are also at higher risk for LUTS [27,28,29,30]. Moreover, higher serum levels of C-reactive protein levels (and thus systemic inflammation) and proinflammatory cytokines are found in both men and women with urinary dysfunction [31]. It can also be local. For example, LUTS caused by BPH is, at least partly, due to prostate inflammation [32]. Or, it may arise in the bladder itself in cases such as UTIs and bladder outlet obstruction. Recently, great strides have been made in understanding the mechanisms underlying this inflammation and such inflammation is known to drive pathological changes such as denervation [33], fibrosis [34] and muscle hypertrophy [35,36] that can ultimately cause decompensation and incontinence. Given this understanding, we will review some of this work in later sections. However, less is known about the role inflammation plays in causing depression.

### 6.2. Neuroinflammation Is Involved in the Etiology of Depression

Evidence to support a role for inflammation in the etiology of depression comes from the many diseases of chronic inflammation listed as known co-morbidities in depressed patients [37]. As mentioned, patients with systemic inflammation due to obesity, diabetes or autoimmune diseases are more likely to experience depression and anxiety [27,28,29,30]. The association of pro-inflammatory co-morbidities with psychiatric disease is bolstered by the fact that depressed patients have increased levels of pro-inflammatory markers such as IL-6 and C-reactive protein in the blood and cerebrospinal fluid [38]. Such correlations between systemic inflammation and depression were intriguing but they led to the observation that depression was actually associated with neuroinflammation within the CNS itself. For example, PET imaging of depressed patients shows neuroinflammation in their prefrontal and anterior cingulate cortices and the hippocampus, as denoted by increased populations of activated microglia [39]. Several chronic inflammatory conditions associated with depression, such as Alzheimer’s or Parkinson’s disease, represent primary CNS inflammation while others (obesity, diabetes, rheumatoid arthritis and psoriasis) have their initial inflammatory processes occurring in the periphery [40]. Thus, it seems that inflammation can lead to mood disorders through an “inside” the CNS mechanism or an “outside to inside” the CNS pathway mechanism [41]. Given its relevance to LUTS-induced mood disorders, we will devote an entire section at the end of this review to discussing possible pathways that drive the “outside to inside” mechanism. However, when considering the bidirectionality discussed above, we wish to point out that the “inside” mechanism might also be considered an “inside to outside” mechanism.

In the 1990s, investigations into “sickness behavior” provided insight into how inflammation can produce alterations in behavior consistent with depression [42,43]. These studies typically involved an immune challenge, often the administration of exogenous cytokines or an inciting agent such as lipopolysaccharide (LPS), to mimic the body’s response to infection or sepsis. Such treatments elicit “sick behavior” in the short term such as a decrease in activity, an increase in sleepiness and a reduction in food and water intake. This behavioral response is adaptive in the acute phase, where conserving energy and shifting metabolism to healing/defense can help defend against infection or heal from an injury. Several investigators found that these challenges produce an inflammatory response within the brain and this response was thought to cause the sick behavior [44,45,46]. In a short-term illness inflammation is soon resolved and neuroinflammation wanes along with the behavioral changes. However, in chronic conditions such as diabetes, the persistent neuroinflammation and chronic sickness behavior becomes maladaptive and precipitates or contributes to chronic psychiatric disease such as depression.

One of the more overwhelming pieces of evidence suggesting inflammation as an etiology for depression lies in the antidepressant effects of a wide range of anti-inflammatory drugs. Three meta-analyses have been written in the past five years that included randomized control trials investigating the effectiveness of anti-inflammatory agents in treating depression [47,48,49]. The range of drugs studied was impressive, including cytokine inhibitors, NSAIDs, omega-3 fatty acids, statins, and other commonly used anti-inflammatory agents. Remarkably, a clinical improvement in depressive symptoms was observed for almost all of the drugs studied, with very few exceptions. Although none of these medications demonstrated a level of efficacy comparative with serotonin reuptake inhibitors (SSRIs—the gold standard treatment for mood disorders), there do appear to be subpopulations who may stand to especially benefit from this approach to therapy. Conversely, it has been recently shown that standard treatments for major depressive disorder (MDD) such as fluoxetine (Prozac—a common SSRI), also have anti-inflammatory properties [50]. This overlap in effects between drug classes once again underscores the intimate relationship between inflammation and mood disorders and their bidirectionality.

## 7. Modern Theories of Depression and the Role of Neuroinflammation

The monoamine hypothesis, which attributes the cause of depression to a deficiency in serotonin, has guided the development of antidepressant therapies for decades [51]. While the success of SSRIs does validate this model, it is important to note that many treatment failures occur with this heterogeneous disease, suggesting that serotonin deficiency alone is a simple but incomplete explanation for the etiology of depression. Other work has found causative alterations in the hypothalamus–pituitary–adrenal (HPA) axis, neurogenesis and neuroplasticity in creating mood disorders. Over the past two decades, neuroinflammation has been added to this list of instigating factors [52]. Moreover, strong evidence suggests that inflammation within the brain can alter the populations of monoamine neurotransmitters, dysregulate the HPA axis and negatively affect neurogenesis and neuroplasticity in the hippocampus and other regions of the brain. Thus, the immune system can directly or indirectly affect major pathways associated with depression in humans. We will briefly review these pathways and how they are affected by inflammation.

### 7.1. Serotonin

The serotoninergic hypothesis of depression is based on the findings that cerebral serotonin concentrations are lower in patients with MDD and that most drugs with antidepressant effects have the ability to raise cerebral serotonin levels [51]. Serotonin is, of course, a very important neurotransmitter in the brain and tryptophan is the essential precursor. However, tryptophan is also a critical nutrient required by bacteria when they invade a human host so, as an early part of the infectious inflammatory response, tryptophan metabolism is upregulated to deplete this important nutrient and essentially starve the pathogen [53,54]. The unfortunate result is a reduction in serotonin levels in the brain. There is also another critical pathway that works to decrease cerebral serotonin levels, particularly in conditions of sterile inflammation, and that is a shift of tryptophan metabolism from serotonin towards kynurenine. Normally the enzyme tryptophan hydroxylase catalyzes the synthesis of most tryptophan to serotonin. However, tryptophan can also be converted to kynurenine by indoleamine 2,3-dioxygenase (IDO). IDO exhibits low activity under most conditions but is activated by pro-inflammatory cytokines such as IL-1β, IFN-α, IFN-γ, TNF-α, and PGE-2 [55]. Increasing IDO activity shifts tryptophan conversion to kynurenine, thus decreasing the amount available to be converted to serotonin. This dearth of serotonin stymies neurotransmission and is thought to be at least partly responsible for the depressive behavior exhibited in response to pro-inflammatory stimuli.

In addition to the decrease in serotonin synthesis, the shunting of tryptophan to the kynurenine pathway has another consequence that might contribute to the development of depression. In particular, several metabolites are produced that have excitotoxic properties including 3-hydroxy-kynurenine, 3-hydroxy-anthralinic acid and quinolinic acid [56]. In concert with other metabolites these products are neuroprotective, but on their own they are neurotoxic and appear to be at least partially responsible for morphological reductions in the prefrontal cortex thickness in MDD patients [57]. These metabolites have also been found to be elevated in many subtypes of depression [58]. Most interestingly, they are also increased in response to immune challenges such as interferon therapy for cancer patients or in preclinical models using LPS [59].

Together, the evidence for an important role for serotonin in depression is long-established and incontrovertible. However, as discussed, conventional studies clearly point to the ability of inflammation to exacerbate these effects in complex and interrelated ways. Thus, the link between inflammation and the decrease in available cerebral serotonin is becoming a hallmark of depression.

### 7.2. The HPA Axis

The second major correlate of MDD is dysregulation of the HPA axis which disrupts the negative feedback and leads to hypersecretion of glucocorticoids [60]. The HPA axis is a major neuroendocrine regulator of homeostasis that responds to stressors via its control over metabolism, the immune system and the autonomic nervous system. Implementation of a full-scale systemic immune response utilizes approximately 25–30% of the basal metabolic rate [61,62] and is therefore a significant stressor to the organism when activated. In this setting, pro-inflammatory cytokines produced either in the brain or in the periphery can act on cells within the hypothalamus to activate the HPA axis and stimulate glucocorticoid release from the adrenal glands [60]. This results in increased energy production (release of glucose from liver, glycogenolysis, lipolysis, etc.) and the development of sickness behavior that decreases energy expenditure from non-essential activities. In the case of actual acute infection, this allows more energy to be dedicated to fighting infection. The behavioral manifestations typically attributed to sickness behavior overlap considerably with those seen in major depressive episodes and are suggestive of the interdependence of inflammation, the HPA axis and depression.

Between 35% and 65% of patients suffering from MDD have HPA axis abnormalities as measured by enhanced glucocorticoid release and/or increased expression of adrenocorticotropic hormone (ACTH) [63]. They have been found to have higher glucocorticoid levels in the plasma, saliva and urine compared to non-depressed patients. While not entirely understood, it appears that this observation is dependent on the changes in the expression and sensitivity of glucocorticoid receptors which short-circuits the negative feedback mechanism [64]. 

Considering the well-established anti-inflammatory action of glucocorticoids, their involvement in the bidirectional relationship between inflammation and depression may seem paradoxical at first. While it makes sense that inflammation can serve as a source of stressor that activates the HPA axis, the converse is not as obvious until one considers that glucocorticoids actually have pro-inflammatory potential [65]. In fact, over the past two decades, several pro-inflammatory effects have been discovered. For example, while glucocorticoids have generally been associated with attenuation of Toll-like receptor (TLR) activation, it is now understood that they can activate TLRs as well, which is an important initiator of the innate immune response [66]. In addition, glucocorticoids enhance inflammatory responses triggered by ATP, an important activator of the innate immune response [67]. In a human endothelial cell line, this was shown to be effectuated by an increase in P2Y2 receptors which increased production of the proinflammatory cytokine IL-6. Perhaps the most central target of ATP in the innate immune system is NLRP3, a protein dubbed the central processing unit of inflammation [68] that plays a major role in triggering inflammation in both the CNS and the periphery (see below for a more detailed description of this critical mediator). Glucocorticoids can act via NF-κβ to promote expression of NLRP3 and enhance inflammation [69]. These proinflammatory effects of glucocorticoids are normally balanced with anti-inflammatory effects, but under conditions of stress, the pro-inflammatory effects can be favored. This may explain how HPA axis hyperactivity can coexist with inflammation in MDD patients who have high levels of both corticosteroids and inflammatory mediators/markers such as IL-1β, IL-6, TNF-α, and activated microglia [70].

### 7.3. Neurogenesis

In the CNS, adaptable responses to inflammatory or stressful stimuli require that functional connectivity between cortical and sub-cortical centers respond appropriately and this process is dependent on neurogenesis [71]. In the adult, neurogenesis occurs primarily in the subventricular zone of the lateral ventricles and the sub-granular zone of the dentate gyrus of the hippocampus. There, it is critical in the development of new synapses and neurons that allow for plasticity in the neural circuitry [72]. Several observations show that inhibition of neurogenesis may be an important pathophysiological event in the emergence of depression. Both animal models of depression and depressed human patients have decreased hippocampal neurogenesis which is reversed by chronic administration of antidepressant drugs [73]. Perhaps more telling is that experimental stimulation of hippocampal neurogenesis protects against depression brought on by stress [74] whereas experimentally decreasing neurogenesis increases signs of depression [75]. Finally, when neurogenesis is ablated, the effectiveness of antidepressant therapy is blocked [76]. Thus, it appears that impairment of neurogenesis may be the third major biological factor in the pathophysiology of depression.

Neuroinflammation, instigated from a central or peripheral source, induces a decrease in hippocampal neurogenesis via activation of microglia in the brain. Immune challenges with LPS activate microglia whose normal role is to phagocytize adult neurons and neuroblasts [77]. Once activated, the microglia secrete several pro-inflammatory cytokines including IL-1β, IL-6, TNF-α and IFN-α, [78]. While the exact mechanism by which these mediators inhibit neurogenesis is not entirely clear, it has been found that IL-1β is directly inhibitory and is an important regulator of proliferation and differentiation of neural precursor cells [79]. IL-1β is processed to its mature form via the activation of the NLRP3 inflammasome which is a critical mediator of neuroinflammation, as will be discussed subsequently.

### 7.4. Towards a Unified Model of Depression

Any contemporary model of depression must consider the major biological factors of serotonin, the HPA axis and neurogenesis, while incorporating the inducing or modulatory effects of inflammation. Even in cases where one biological instigator is primarily responsible for the state of depression, it is likely that most, or all, of the major effectors will be altered and as such contribute to or mitigate the depression. Given the recent appreciation of a role for inflammation, it is essential that it be assimilated in any modern comprehensive model. Only such assimilation can lead to an understanding of the bidirectional link between inflammation and depression.

## 8. The Mechanism by Which Inflammation Is Triggered—The NLRP3 Inflammasome

**Figure 1:**
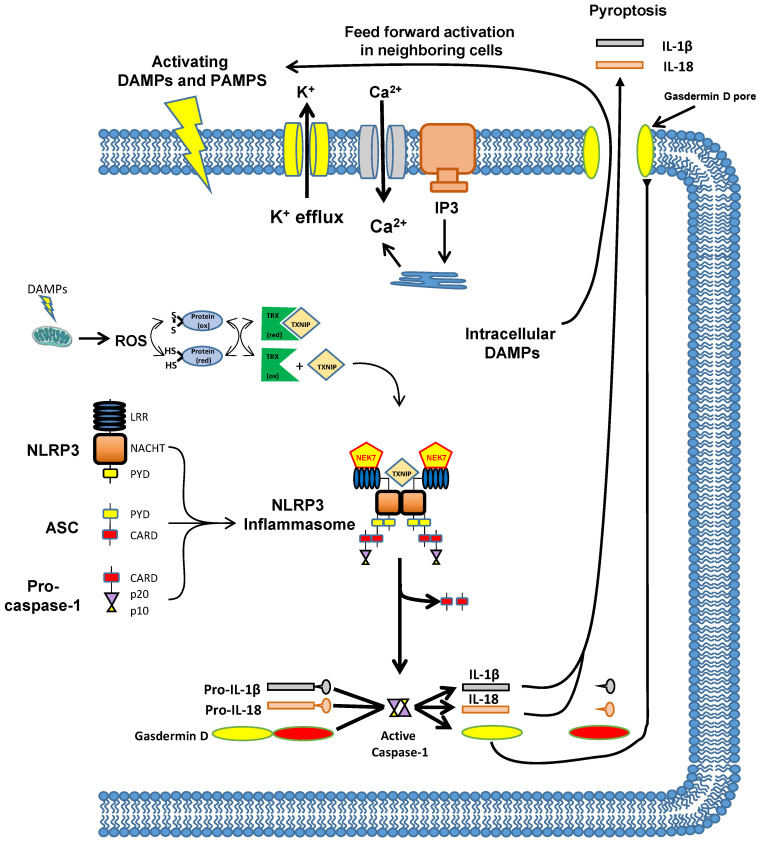
Illustration of the NLRP3 inflammasome, pathways of activation and biochemical mechanism of action. As described in the text, the NLRP3 inflammasome can be activated by DAMPS and PAMPS in a multitude of ways, mostly illustrated at the top membrane. DAMPs may also alter release of ROS which free TXNIP to stabilize a developing NLRP3 oligomer. Upon activation, the NLRP3 inflammasome assimilates and activates caspase-1 which cleaves pro-IL-1β and pro-IL-18 into their active forms. It also cleaves Gasdermin D and the N-terminus forms a pore in the plasma membrane. This pore facilitates release of IL-1β and IL-18 to act locally and systemically as a pro-inflammatory cytokine. The Gasdermin D pore also releases intracellular DAMPS which can affect neighboring cells to trigger further NLRP3 activation in a feed-forward mechanism. This figured was modified, with permission from Wolters Kluwer, from [81] and incorporates portions from [80] (open access).

Before delving deeper into the importance of inflammation in various diseases, it is useful to briefly review the modern concept of how most inflammation is triggered; through structures known as inflammasomes. The innate immune system recognizes molecular patterns, as opposed to specific antigens. These patterns are known as pathogen-associated molecular patterns (PAMPS) when associated with invading pathogens (e.g., LPS) or damage (danger) associated molecular patterns (DAMPS) when released from inside cells in response to physical injury (e.g., ATP), or produced by deranged metabolism such as during diabetes (e.g., monosodium urate, advanced glycation end products (AGEs), etc.). DAMPS and PAMPS are recognized by five classes of pattern recognition receptors (PRRs); Toll-like receptors (TLRs), c-type lectin receptors, dsDNA receptors, retinoic acid-induced gene-I (RIG-1)-like receptors and the Nucleotide-binding oligomerization domain (NOD)-like receptors (NLRs). These receptors primarily promote gene expression and Toll-like receptors (TLRs) are particularly well known for promoting transcription of proinflammatory cytokines through NF-κB. The primary exception is the NLRs, most of which form macromolecular structures called inflammasomes after activation. An additional exception is AIM2, a dsDNA receptor that also forms an inflammasome. The inflammasomes are named for the NLR involved and most recognize specific PAMPS and thus are involved in infectious inflammation. However, NLRP3 recognizes a multitude of DAMPS and seems to mediate most instances of sterile inflammation. This inflammasome and its actions are illustrated in Figure 1 [80]. In the inactivate state, NLRP3 is a monomer with three domains: a N-terminal pyrin (PYD) domain that mediates homotypic binding, a nucleotide-binding and oligomerization (NACHT) domain that mediates ATP-dependent oligomerization and a C-terminal leucine-rich repeat (LRR) domain that senses ligand (most likely indirectly in the case of NLRP3). While the exact mechanism of activation is hotly debated, it may involve several pathways that may differ for different DAMPS. Indeed, there are canonical, noncanonical and alternative pathways of activation (for review see [81,82,83,84,85,86]), but common themes are K^+^ efflux across the plasma membrane, increases in intracellular Ca^2+^ release across the plasma membrane or from the ER, and increased reactive oxygen species (ROS) produced from the mitochondria. The latter is particularly interesting as the ROS is known to oxidize intracellular proteins. In turn these proteins are acted upon by reduced thioredoxin (TRX), restoring the proteins while oxidizing the TRX. In the reduced state, the TRX is associated with thioredoxin-interacting protein (TXNIP) and upon oxidization TXNIP dissociates from TRX and is then free to interact with the inflammasome components where it promotes the formation of an oligomer.

Once activated, NLRP3 is deubiquitinated, licensing it to form the oligomer. For this, NACHT domains of adjacent NLRP3 molecules associate through homotypic binding, with structural roles for both TXNIP and another protein called never in mitosis gene a-related kinase 7 (NEK7). This facilitates homotypic binding between the pyrin domain on NRP3 and a pyrin domain on an adaptor protein known as apoptosis-associated speck-like protein containing a CARD (ASC). ASC also contains a caspase recruitment domain (CARD) and once ASC is bound to NLRP3 this domain binds a CARD on procaspase-1. While Figure 1 shows only a dimer for simplicity, the NLRP3 forms large oligomeric structures through these homotypic domains. Binding of multiple molecules of procaspase-1 results in their cleavage and activation through a process known as induced proximity. Cleavage releases two important subunits, P10 and P20. These associate with an additional set of P10 and P20 to form the active tetramer. Active caspase-1 then acts upon pro-IL-1β and pro-IL-18 to cleave them into their mature and active forms. Caspase-1 also cleaves a protein called Gasdermin D and the N-terminus of Gasdermin D translocates to the plasma membrane and forms a pore. The pore then allows the release of the mature IL-1β and IL-18 through a programmed necrotic process called pyroptosis. Pyroptosis also has the effect of releasing intracellular DAMPS out into the extracellular environment where they can proceed to activate NLRP3 in neighboring cells and thus create a feed-forward cascade triggering massive inflammation in the tissues. It is through this mechanism that the NLRP3 inflammasome forms and triggers inflammation in many different cell types including in peripheral organs and the CNS.

## 9. Pro-Inflammatory Events in Peripheral Organs or Tissues Result in Concurrent Inflammatory Changes in the CNS

The concept that inflammation in the lower urinary tract may lead to inflammation in the brain and resulting mood disorders is rooted in the precedent discovered in other diseases characterized by peripheral inflammation. Here, we briefly review several of these diseases before discussing evidence emerging from the bladder literature and ending with current ideas as to how peripheral inflammation is transferred to the CNS.

### 9.1. Inflammatory Bowel Disease (IBD)

Possibly the greatest evidence for peripheral insults eliciting neuroinflammation and depression comes from inflammatory bowel disease (IBD) [87] where mood disorders can affect up to 33% of patients [88]. Unfortunately, depressive symptoms persist even when the disease is in remission [89]. One of the defining characteristics of IBD is inflammation in the gut and animal models are providing great insight into how this translates into CNS pathology. For example, colitis induced by dextran sodium sulfate in drinking water induced altered microglial phenotypes within the CNS [90,91]. There was also a decrease in tight junction proteins (occludin and claudin-5) in the cortex and hippocampus [91] clearly indicating damage to the blood–brain barrier (BBB). Long-term models show severe pathology in the colon along with significant upregulation of pro-inflammatory mediators such as NLRP3, caspase-1, and IL-1β [92] in the cortex and hippocampus. NLRP3 was upregulated in both astrocytes and microglia in these studies. There was also a significant decline in hippocampal neurons likely reflecting, at least in part, a decrease in neurogenesis. This loss of neurons may explain the persistence of depressive symptoms in patients who are in remission, as well as the decline in cognitive function in experimental mice. Interestingly, when colitis was induced in NLRP3-deficient mice, inflammatory pathology was absent in both the gut and brain [92], clearly identifying a central role for this structure and the inflammation it triggers.

### 9.2. Rheumatoid Arthritis

Rheumatoid arthritis (RA) is another chronic inflammatory condition associated with a high incidence of depressive symptoms [93,94,95]. In humans it is well known that RA increases systemic pro-inflammatory cytokines but it also upregulates pro-inflammatory IL-1β in the cerebrospinal fluid [96] while decreasing homeostatic microglia [97], both of which signify inflammation in the CNS. In animal models, there is a clear breakdown of the blood–brain barrier and barrier damage corresponds to a decrease in tight junction proteins occludin and ZO-1 [98]. Moreover, activated microglia in the cerebral cortex and hippocampus of arthritic mice increased by over 200% [99] while showing significant upregulation of IL-1β and TNF-α, again signs of inflammation in the CNS. Within the cortical and hippocampal regions, the number of CD206 (a macrophage mannose receptor present on peripherally activated macrophages)-positive cells was markedly increased suggesting the migration of activated macrophages into the brain from the periphery occurs due to a disrupted blood–brain barrier [99]. Such mechanisms by which peripheral inflammation affects the CNS are discussed more in depth in a subsequent section.

### 9.3. Sepsis

Sepsis is characterized by systemic inflammation and multiple organ dysfunction. In humans receiving an inflammatory challenge that mimics sepsis (LPS injection), there was the expected increase in sickness behavior along with enhanced levels of circulating pro-inflammatory cytokines, but positron emission tomography (PET) also revealed microglial activation [100]. Studies using animal models have shown that systemic administration of LPS causes significant upregulation of IL-1β, IL-6 and TNF-α in both the brain and spinal cord [101] as well as microglia activation. As microglia become activated, changes in cell metabolism occur leading to increased oxidative stress which in turn triggers NLRP3 activation, pyroptosis and release of IL-1β and IL-18 which further activate NLRP3. Consequently, microglial and NLRP3 activation is associated with the severity of sepsis-associated encephalopathy.

## 10. Animal Models Confirm the Connection between Bladder Inflammation, Neuroinflammation and Mood Disorders

**Figure 2:**
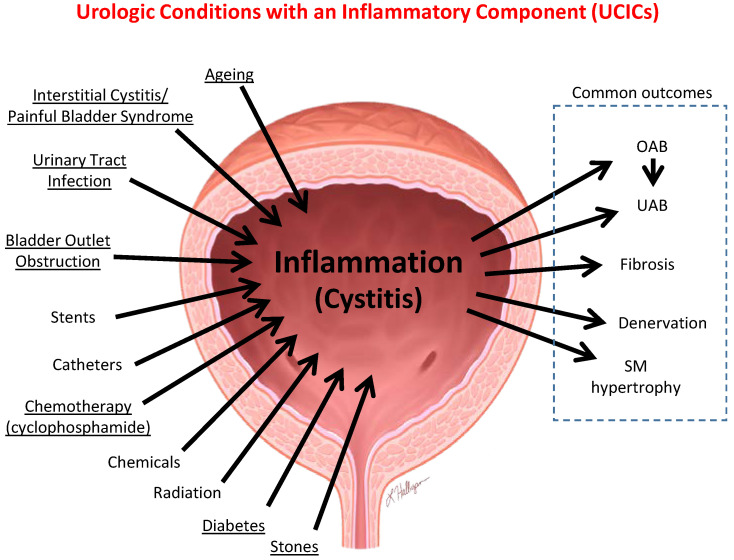
Illustration of the numerous Urological Conditions with an Inflammatory Component (UCICs). There are a number of conditions in the bladder that have been shown to be associated with inflammation, either as an initiating etiology or a contributing factor. These are illustrated with inwardly pointing arrows. Many of these are associated with common outcomes indicated by outgoing arrows and surrounded by a dashed box. Not all outcomes are associated with all UCICs. NLRP3 in the bladder has been shown to play a critical role in the underlined conditions. Some of these conditions have been associated, directly or anecdotally, with mood disorders.

While a clinical association between LUTS and mood disorders such as depression seems apparent, recent studies in rodent models have begun to tie this directly to inflammation in the brain. It is hoped that discoveries in these models are applicable to the human situation and may guide treatment paradigms. There are numerous conditions in the bladder that have an inflammatory component associated with them, either as an initiating etiology or a contributing factor. Some of these conditions are shown in Figure 2 and are dubbed Urologic Conditions with an Inflammatory Component (UCICs). Incidentally, NLRP3 within the bladder has been shown to play a major role in most of these disorders (underlined Conditions in Figure 2, it has not yet been looked at in the others). While there may be anecdotal evidence that many of these conditions effectuate or exacerbate psychiatric disorders, recent basic studies in two models have begun to shed insight into the mechanisms by which this may be occurring.

### 10.1. The Cyclophosphamide-Induced Hemorrhagic Cystitis Model

Cyclophosphamide is a common chemotherapeutic drug that is metabolized to acrolein and stored in the urine prior to secretion. There it causes damage to the urothelia and elicits a massive inflammatory response resulting in hemorrhagic cystitis [102]. While hemorrhagic cystitis historically posed significant clinical complications to chemotherapy, its effects are minimized at present through concomitant treatment with hyperhydration and administration of 2-mercaptoethane sulfonate sodium (Mesna), which binds to and masks the effects of acrolein in the urine. However, because of the extensive and rapid response in the bladder (massive inflammation only 24 h after a single injection) the model remains in widespread use today for basic scientists where it primarily serves as a speedy proof-of-concept model for investigating novel pathways and consequences of bladder inflammation. For these reasons, we chose it for our first model to investigate the effect of bladder information on mood disorders [10]. While the model works equally well in rats and mice, we initiated these studies in rats. Importantly, neither cyclophosphamide, acrolein nor any of its metabolites cross the blood–brain barrier [103,104] where they might complicate the mechanisms under study. Additionally, injury to the gut is well-known to trigger neuroinflammation, as just discussed, so it was important that CP does not inflame the gut and thus secondarily alter neuroinflammation. In fact, CP is actually used as an anti-inflammatory treatment for inflammatory bowel diseases [105], minimizing this concern.

**Figure 3:**
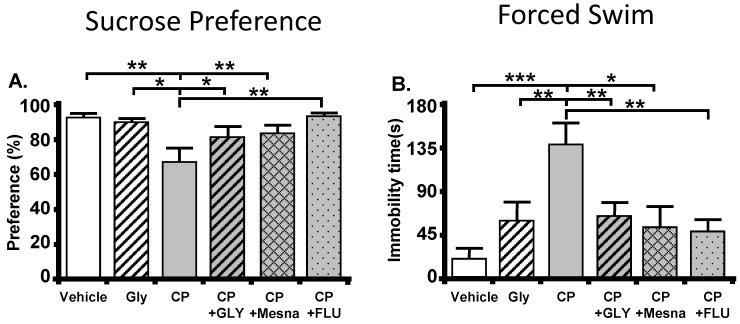
CP induces behavioral signs of depression through NLRP3. (**A**). Sucrose preference test. A reduction of preference indicates depression. Bars represent the mean ± SEM. [Vehicle: *n* = 10; GLY: *n* = 4; CP: *n* = 8; CP + GLY = 6; CP + Mesna = 12; GP + FLU=8]. * *p* < 0.05 and ** *p* < 0.01 by one-way ANOVA and Student-Newman-Keuls post-hoc analysis. (**B**). Forced Swim assay. An increase in time spent immobile indicates depression. Bars represent the mean ± SEM. [Vehicle: *n* = 9; GLY: *n* = 18; CP: *n* = 8; CP + GLY = 18; CP + Mesna = 6; GP + FLU = 11]. * *p* < 0.05, ** *p* < 0.01 and *** *p* < 0.001 by one-way ANOVA and Student-Newman-Keuls post-hoc analysis. This figured was reprinted, with permission from the American Physiological Society, from [10].

For these studies [106], we began assessing signs of depressive behavior 24 h after an i.p. injection of CP (150 mg/kg), although the assays took an additional 24–48 h to complete. Here, we used two common and independent assays of depression, the sucrose preference assay and the forced swim assay. The sucrose preference assay quantitates the rat’s choice of drinking sucrose-laden water versus plain water and is considered an assay of anhedonia, or the inability to experience pleasure (a common symptom of depression). The forced swim assay places the rat in an inescapable container of water and quantifies the amount of time the rat “gives up” trying to escape and just floats. This is an assay of hopelessness or despair, another common sign of depression. Rats given CP show a decreased preference for sucrose (Figure 3A) and an increase in immobility in water (Figure 3B). Moreover, these effects were reversed with glyburide, an inhibitor of the NLRP3 inflammasome and resulting inflammation. Two important controls were included as well. One was pretreatment with Mesna, which acts in the bladder to mask acrolein. This blocked the depression signs indicating the response was directly attributable to the acrolein-induced events in the bladder. Second, we pretreated rats with the antidepressant fluoxetine to demonstrate that CP triggers true depression and not a sick behavior, as sick behavior is not affected by antidepressant. Fluoxetine also blocked signs of depression indicating true depression in these rats.

**Figure 4:**
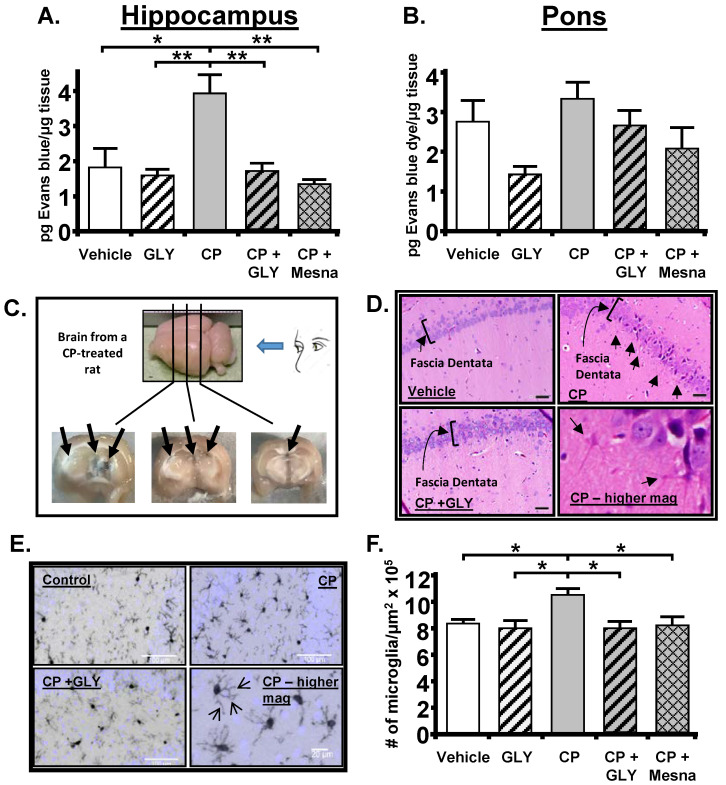
CP-induced cystitis results in inflammation and breakdown of the blood brain barrier in the hippocampus, not in the pons. Administration of GLY or Mesna blocks this effect. (**A**). Evans blue extravasation was increased in the Hippocampus by CP. This increase was prevented by treatment with Gly or Mesna. All results were calculated as pg of Evans blue per μg of tissue. Bars represent mean ± SEM. For A: Vehicle: *n* = 3; GLY: *n* = 3; CP: *n* = 4; CP + GLY: *n* = 4; CP + Mesna: *n* = 4. For B: Vehicle: *n* = 4; GLY: *n* = 3; CP: *n* = 4; CP + GLY: *n* = 4; CP + Mesna: *n* = 8. * *p* < 0.05, ** *p* < 0.01 by one-way ANOVA and Student-Newman-Keuls post-hoc analysis. (**B**). Evans blue extravasation was not significantly changed in the pons by any treatment. (**C**). CP-induced cystitis results in areas of gross blood brain barrier breakdown, with Evans blue dye apparent (arrows) in the periventricular region of the hippocampus. A CP-treated rat was injected with Evans blue as described in the Methods section. After 1 h the brain was removed, sectioned coronally with a scalpel at the approximate locations indicated and photographed. (**D**). CP results in an NLRP3-dependent increase in number of microglia-like cells within the fascia dentata of the hippocampus. Coronal sections (10 µm) were cut through the hippocampus and an H&E stain was performed using routine methodical techniques. Slides were visualized at 60×. Activated glial cells are indicated by arrows. (**E**). Immunohistochemistry shows increased density of activated microglia within the fascia dentata. Coronal sections (10 µm) were cut and Immunohistochemistry was performed using an anti-IbA1/AIF1 antibody and routine histological methods. Slides were visualized at 20× and the number of microglia was quantitated. Arrows demonstrating increased glial processes (arrows) at higher magnification are shown. (**F**). Density of Microglia. Results are depicted as the number of microglia per μm^2^. Bars represent mean ± SEM. [Vehicle: *n* = 5; GLY: *n* = 6; CP: *n* = 7; CP + GLY: *n* = 4; CP + Mesna: *n* = 8]. * *p* < 0.05 by one-way ANOVA and Student-Newman-Keuls post-hoc analysis. This figured was reprinted, with permission from the American Physiological Society, from [10].

Next, based on the discussions above, we examined neuroinflammation in these rats, focusing on two main areas; the hippocampus, because of its known association with depression, and the pons, due to its well-known function in micturition via the pons micturition center. For this, we used the Evans blue dye extravasation assay which measures the movement of dye across the endothelia and is an indication of the extravasation potential of immune cells in the tissue examined [106,107,108,109]. Moreover, when considering the blood vessels traversing the brain, this assay also serves as a sensitive marker of the integrity of the blood–brain barrier [110,111]. What we found was a significant increase in dye permeability into the hippocampus (Figure 4A) but not the pons (Figure 4B). This was blocked by glyburide and Mesna, and thus was dependent on the NLRP3 inflammasome and triggered by acrolein in the bladder. The influx of dye was actually visible in gross cross sections (Figure 4C) and could be localized to the periventricular region of the hippocampus in an area of true blood–brain barrier breakdown and not through the circumventricular organs. The neuroinflammation was also apparent in H&E stained slides as cells with the morphology of activated microglia, primarily in and around the fascia dentata region (Figure 4D). We confirmed this finding by staining for IbA1/AIF (a marker of activated microglia), and quantitating the density of these cells (Figure 4E,F). The density of activated microglia was increased by CP and this was again blocked by both glyburide and Mesna. Overall, this was the first experimental study to show that an insult to the bladder could cause depression in an animal model and that depression was mediated by triggering distant inflammation in the hippocampus.

Unfortunately, there are significant limitations with the CP model. For example, the inflammation caused by CP results in significant bladder pain, in fact CP treatment is often touted as a model of interstitial cystitis/bladder pain syndrome (IC/BPS), and pain itself can bring about depression [26], which can make interpretation difficult. However, following our study the CP model was linked to neuroinflammation in the hippocampus in an additional study [112], although most reports have focused on the spinal dorsal horn [113,114,115]. In addition, bladder pain may limit mobility in the forced swim model of despair, leading to inappropriate or enhanced measurements of depression, although we think this is unlikely since the anti-depressant fluoxetine restored mobility but would not have alleviated the pain. In addition, CP is often studied for its contribution to chemotherapy-induced cognitive impairment (“chemo brain” in layman’s terms) [116,117]. While the mechanisms may, or may not, be the same, these associations can complicate interpretations. This model is further limited by its acute nature whereas depression or anxiety are chronic conditions. Thus, studies on the effects of bladder inflammation on depression/anxiety should be more accurately represented with chronic models. In addition, thanks to Mesna, CP-induced hemorrhagic cystitis is a relatively rare occurrence in humans so conclusions drawn on the model have limited clinical scope. Therefore, we sought to investigate these concepts in a clearly bladder-centric chronic disease model with a significant clinical correlate—bladder outlet obstruction (BOO).

### 10.2. The Bladder Outlet Obstruction (BOO) Model

BOO occurs most commonly in patients with benign prostate hyperplasia (BPH), which affects approximately 70% of men over the age of 70 and costs nearly USD 4 billion annually in the USA [118]. However, it can also result from other conditions such as stones, organ prolapse in women and posterior urethral valves in children. While each of these conditions has associated co-morbidities and therefore likely activate a myriad of pathways, we can isolate the effect on the bladder by recapitulating the obstruction surgically with a circumferential suture around the urethra in which a catheter has been placed, then removing the catheter. This allows us to work with a more simplistic system to better define the mechanism of how the obstructed bladder causes mood disorders, without concern for exacerbating (or mitigating) input from secondary pathways.

**Figure 5:**
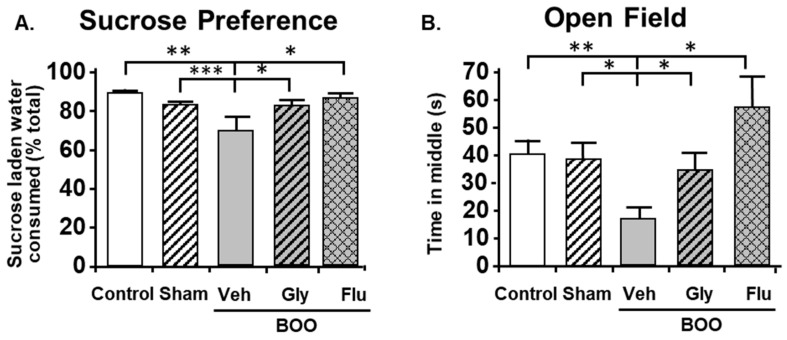
After 12 weeks of BOO, rats show signs of depression. These behavior differences were not present when rats were given glyburide or fluoxetine (Flu), an anti-depressant. (**A**). The sucrose preference assay (a measure of anhedonia). The assay was performed as described in the Materials and Methods section. The results are presented as the amount of sucrose laden water consumed as a percentage of the total volume imbibed. Veh = vehicle-treated, Gly = glyburide-treated. Results are the mean ± 95% confidence intervals; * *p* < 0.05, ** *p* < 0.01 and *** *p* < 0.005 by ANOVA and Dunn’s test. (*n* = 24, 24, 14, 14, 6). This figured was reprinted, with permission from the Wiley Periodicals, from [9]. (**B**). The open field assay (a measure of anxiety.) The assay was performed as described in the Materials and Methods section and scored by a blinded investigator. The results are presented as the time in which at least 2 paws were present in the middle section of the open field during the 10 min test session. Veh = vehicle-treated, Gly = glyburide-treated. Results are the mean ± 95% confidence intervals; * *p* < 0.05 and ** *p* < 0.01 by ANOVA and Dunn’s test. (*n* = 26, 23, 15, 13, 6).

**Figure 6:**
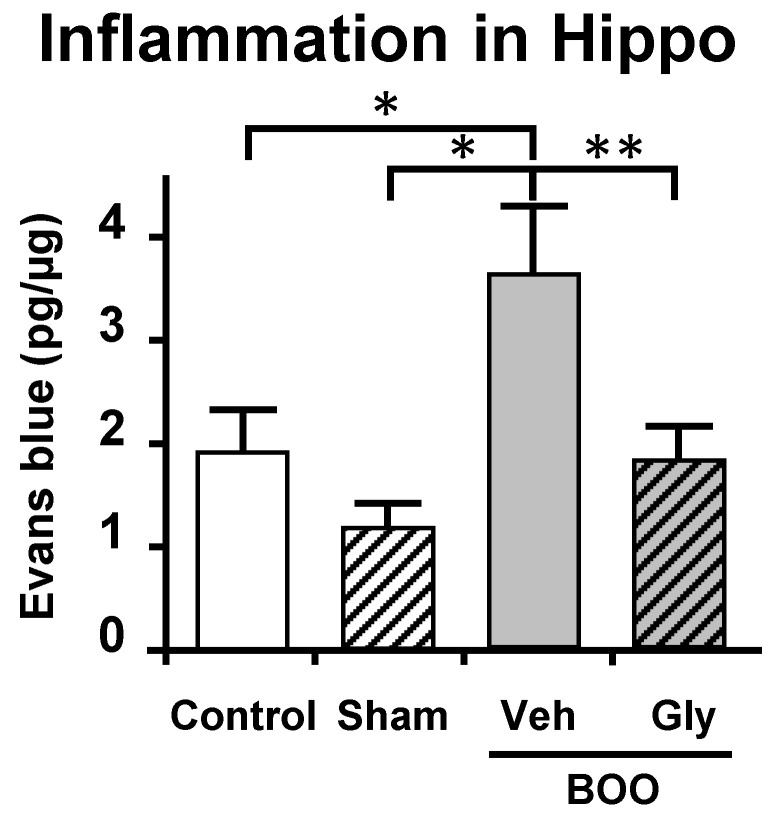
After 12 weeks of BOO, inflammation is present in the hippocampus of rats. This inflammation is blocked by concomitant treatment with glyburide. Following the treatments indicated, inflammation was assessed by the Evans blue assay as described in the Materials and Methods section. Veh = vehicle-treated, Gly = glyburide-treated. Results are the mean ± 95% confidence intervals; * *p* < 0.05 and ** *p* < 0.01 by ANOVA and Dunn’s test. (*n* = 8, 7, 12, 8). This figured was reprinted, with permission from the Wiley Periodicals, from [9].

**Figure 7:**
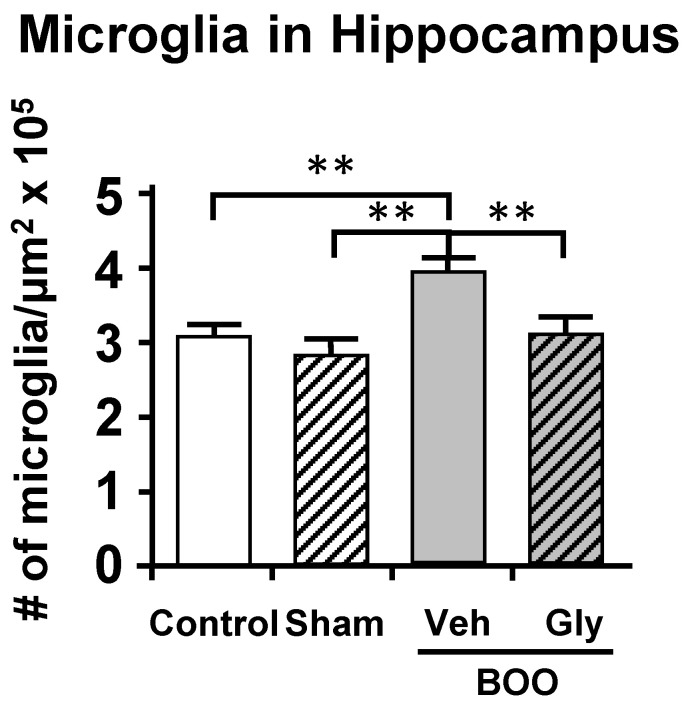
After 12 weeks of BOO the number of activated microglia in the hippocampus is increased and this increase was blocked by glyburide treatment. Activated microglia in 10 µm sections of brain were stained for IbA1/AIF1 and then visualized and quantitated. The results are presented as the density of activated microglia per µm^2^. Veh = vehicle-treated, Gly = glyburide-treated. Results are the mean ± 95% confidence intervals; ** *p* < 0.01 by ANOVA and Dunn’s test. (*n* = 7, 6, 8, 7). This figured was reprinted, with permission from the Wiley Periodicals, from [9].

**Figure 8:**
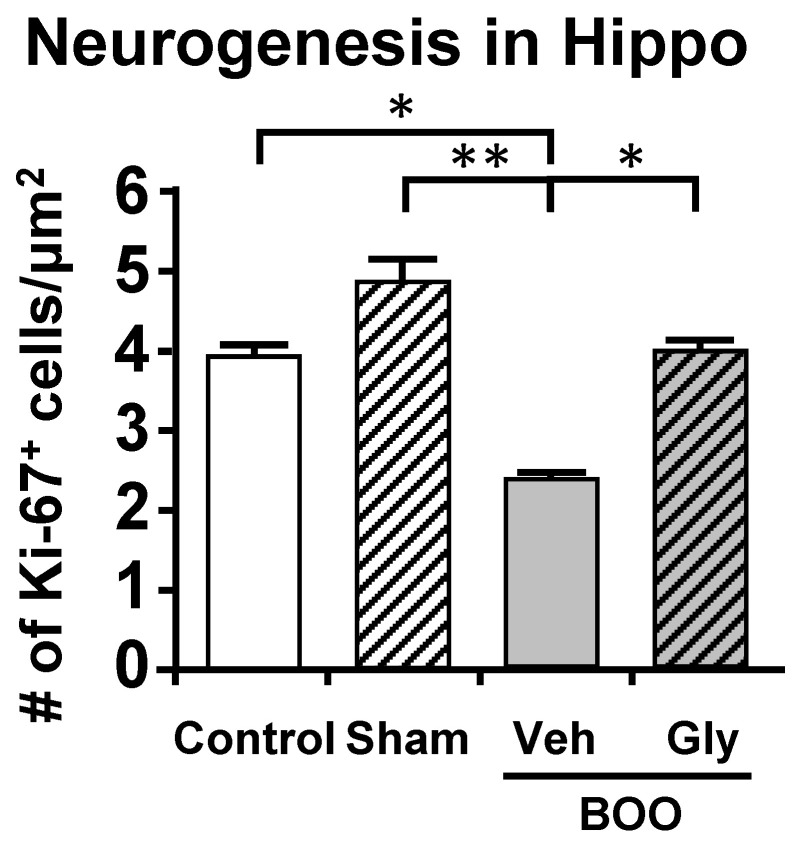
After 12 weeks of BOO, neurogenesis is statistically decreased in the hippocampus and this increase is blocked by glyburide treatment. Cells in 10 µm sections of brain were stained for Ki-67 and then visualized and quantitated as described in the Materials and Method section. The results are presented as the density of Ki-67+ cells per µm^2^. Veh = vehicle-treated, Gly = glyburide-treated. Results are the mean ± 95% confidence intervals; * *p* < 0.5 and ** *p* < 0.01 by ANOVA and Dunn’s test. (*n* = 6, 6, 6, 6). This figured was reprinted, with permission from the Wiley Periodicals, from [9].

For these studies [109], we utilized the sucrose preference assay of anhedonia and the open field assay of anxiety. These assays were thought to minimize effects of pain known to be present in the CP model. Similar to what occurred in the CP model, BOO rats showed a significant decrease in preference for sucrose laden water which was prevented by inhibiting NLRP3-induced inflammation with glyburide or treatment with the antidepressant fluoxetine (Figure 5A). Identical conclusions were made with the open field assays of anxiety where the rat’s propensity to explore the open center areas of a large well-lit box were curtailed by obstruction and maintained/restored with glyburide and fluoxetine, respectively (Figure 5B). Much like the CP model, using Evans blue we were able to detect significant inflammation in the hippocampus of the BOO rats that was blocked by inhibiting NLRP3 with glyburide (Figure 6). This too was associated with an increase in activated microglia (Figure 7). However, in those studies we carried it a step further and looked at neurogenesis in the hippocampus by Ki-65 staining [119]. We found that changes in neurogenesis completely paralleled inflammation and activated microglia levels (Figure 8). However, it is important that these studies required 12 weeks of BOO to detect signs of depression [9], compared to 12 days for bladder inflammation and dysfunction [109] and 6 weeks for bladder decompensation [120]. Thus, signs of depression are clearly only associated with chronic BOO. Moreover, neuroinflammation could be detected as early as 6 weeks [9], which suggests a sequence of events where neuroinflammation leads to depression. Overall, we have shown in a clinically relevant, and clearly bladder-centric, mouse model that inflammatory insults to the bladder directly lead to inflammation in the hippocampus and signs of depression.

## 11. Possible Mechanisms by Which Inflammation in the Bladder Is Converted into Neuroinflammation in the CNS

**Figure 9:**
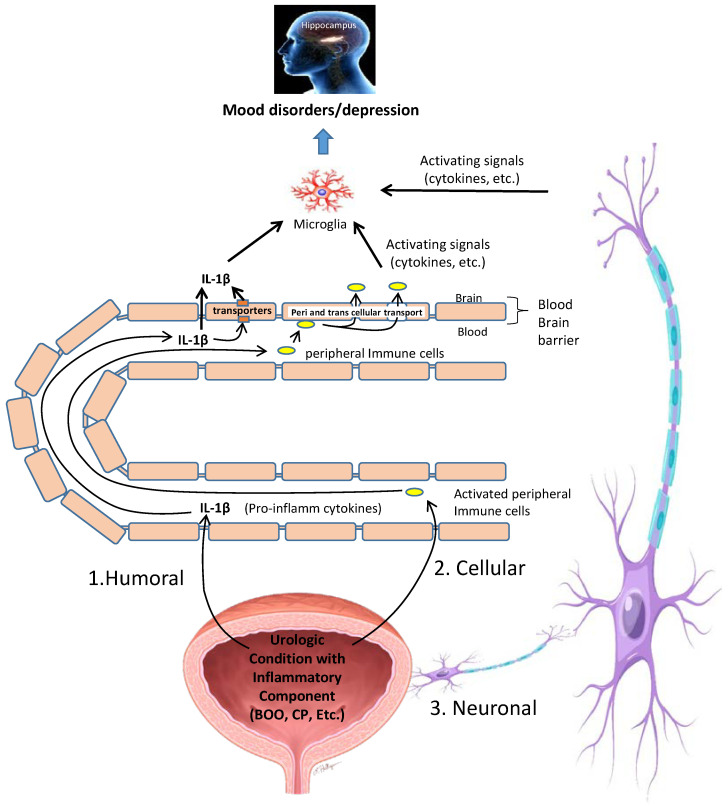
Illustration of possible mechanisms by which inflammation in the bladder caused by a Urinary Condition with an Inflammatory Component (UCIC) is converted into neuroinflammation in the CNS. Depicted are three possible mechanisms that can convey a signal of inflammation into the CNS where its translated into neuroinflammation, perhaps in many areas but most certainly in the hippocampus. 1. The humoral pathway whereby proinflammatory cytokines are released from the bladder and transferred to the brain trough the circulatory system. There they pass through the BBB (either paracellularly or through transporters) and into the brain where they activate microglia (and perhaps astrocytes) to trigger neuroinflammation and mood disorders. 2. The cellular pathway whereby immune cells are activated in the periphery and travel through the circulatory system to the brain where the cross the BBB through extravasation as well as pores in the endothelial cells themselves. Inside the BBB they activate microglia (and perhaps astrocytes), triggering inflammation and mood disorders. 3. The neural pathway whereby activating signals (cytokines, etc.) are carried into the brain through ascending fibers to activate microglia (and perhaps astrocytes) to trigger inflammation and mood disorders. Some image(s) used under license from Shutterstock.com (hippocampus, microglia and neuron).

While the experimental data connecting inflammatory insults in the bladder to inflammation in the hippocampus and depression are clear, the mechanism by which this occurs is not. In fact, the mechanism by which any peripheral insult triggers inflammation in the brain is a hotly debated topic but it clearly involves activating the NLRP3 inflammasome in the brain [121]. Currently, there are three distinct pathways that have been proposed [122] and these are non-exclusive so any given periphery-to-brain model may employ more than one. The foremost theory is the humoral pathway (Figure 9, marked with a #1) that proposes movement of proinflammatory cytokines produced in the periphery, such as Il-1β, directly across the BBB where they trigger additional inflammation in the brain tissue. The contribution of such a mechanism to bladder-induced depression seems probable as we detected breakdown of the BBB in both the CP and BOO models [9,10]. Moreover, our lab, and others, have demonstrated the importance of IL-1β in CP-induced bladder inflammation and even measured increased serum levels of Il-1 β, TNF-α, and IL-6 in the CP model [33,34,123,124]. There are also saturable transport molecules capable of moving peripheral cytokines across the BBB [122], such as the atypical chemokine receptor 1 (ACKR1) and the Duffy antigen/receptor for chemokines (DARC), although roles for these transporters have not been examined in CP or BOO models.

In the second pathway (Figure 9 #2), immune cells are activated as part of the inflammation in the bladder and these immune cells travel to the brain via the circulation before crossing directly through the BBB and propagating inflammation in the CNS. This pathway, called the cellular pathway, is probably best worked out in the popular animal model of multiple sclerosis, experimental autoimmune encephalitis (EAE) [125]. In EAE, high numbers of CD4+ T-cells cross the BBB and have been shown to do so through two different pathways: paracellularly through the complex tight junctions between endothelial cells, and transcellularly via a pore in the endothelial cell body itself [125,126]. The resulting centrally located lymphocytes then either remain active or may recognize their cognate ligands on perivascular antigen presenting cells and become reactivated behind the BBB, consequently stimulating neuroinflammation [125]. Perhaps surprisingly, this cellular pathway may be even more complex as was discovered in studies of the inflamed liver, which releases TNF-α. Peripherally produced TNF-α crosses the BBB and stimulates microglia to release CC-chemokine ligand 2 (CCL2). CCL2 then passes back across the BBB into the periphery and triggers monocyte movement into the brain [127] where they subsequently trigger neuroinflammation. As stated earlier, there are increases in serum levels of TNF-α in response to CP [123], so it is possible this pathway is involved as well. Clearly, there is considerable fodder for future exploration in this area.

Finally, in the third pathway, the neural pathway (Figure 9 #3), afferent neurons are stimulated by proinflammatory cytokines in the periphery which carry the information through ascending fibers into the brain where they are translated back into cytokine signals that activate microglia and astrocytes, triggering neuroinflammation in the CNS [128]. In the CP model, there is some evidence of neural transfer of an inflammatory signal into the spinal cord, if not the brain, as Liu et al. found an inflammatory phenotype in the L6-S1 quadrants of the spine [113]. One can infer additional support for this pathway from the studies of pain associated with CP as peripheral pain is known to stimulate depression and this is most likely mediated through this neural transfer pathway [26].

## 12. Translational Potential

Given the causative relationship between inflammation and depression, there seems to be great translational potential to using anti-inflammatory drugs to assist in treating mood disorders [48,129]. Indeed, numerous classes of anti-inflammatory agents have shown promise in this regard, although none have been tested regarding LUTS-induced mood disorders. For example, nonsteroidal anti-inflammatory drugs (NSAIDs) are, in general, associated with improved antidepressant treatment [130] and, among those, selective COX-2 inhibitors appear to have a more pronounced effect than non-selective COX-2 inhibitors. However, these effects are small and some large randomized controlled trials have not found any significant effects [131]. Cytokine inhibitors, such as the TNF-α inhibitor infliximab, also show potential but their effects are quite modest [131]. Statins are a widely prescribed and well tolerated anti-cholesterol drug with anti-inflammatory properties and several studies have found a decrease in depressive symptoms when statins were taken with fluoxetine, over fluoxetine itself [132,133]. However, some have noted that lowering cholesterol could actually worsen depressive symptoms [134]. Other currently available candidates include corticosteroids [135,136], the antidiabetic drug Pioglitazone [137,138], the antibiotic minocycline [139] and the anti-epileptic drug Modafinil [140,141], but these all carry significant risks of side effects. While it does not appear likely that anti-inflammatory will replace SSRIs for most depressed patients, there may be a small subset of patients who could stand to benefit from them, at least as an adjunct to other medications.

One intriguing possibility would be to engage the cholinergic anti-inflammatory reflex [142,143,144,145]. It is well known that the vagal nerve mediates a cholinergic anti-inflammatory reflex pathway whereby proinflammatory cytokines such as IL-1β (and others) activate the afferent vagal nerves. When the signal arrives in the brainstem, a response signal is propagated through the efferent vagal nerves which generates an anti-inflammatory signal in the periphery. This end signal is best worked out in the splenic nerve where noradrenaline is released from the nerve terminal which is then taken up by memory CD4+ T lymphocytes expressing the beta-2 adrenaline receptor (β2AR) [146]. These cells then release acetylcholine which, in turn, acts through the α7 nicotinic acetylcholine receptor (α7nAChR) on macrophages to reduce production of proinflammatory cytokines (IL-1β, TNBFα, IL-6 and IL-18) and generally act in an anti-inflammatory manner. Depression in animals can be promoted by knocking out or antagonizing α7nAChR [147,148] and acetylcholinesterase levels are increased in depression [149], both of which would reduce the anti-inflammatory properties of this cholinergic pathway and thus enhance inflammation, including neuroinflammation, and depression. However, conflicting literature has alternatively suggested that depression is related to an increase in the cholinergic system and that stimulation of α7nAChR by acetylcholine mediates depression-like behaviors [150,151,152]. Moreover, some suggest that the application of acetylcholinesterase inhibitors may actually lead to depression in Alzheimer’s patients [153].

We believe one class of agents that holds great promise for treating neuroinflammatory depression (including LUTS-induced depression) [154] is the polyunsaturated fatty acids (PUFAs) derived from eicosapentaenoic acid (EPA) and docosahexaenoic acid (DHA) that modulate the resolution of inflammation. In vivo, EPA and DHA are substrates that are turned into molecules known as Specialized Proresolution Mediators (SPMs) (for review see [154,155,156,157,158,159,160]). These are classified as resolvins, maresins, protectins and lipoxins. A fifth class is composed solely of the protein Annexin-A1. These molecules are increased in tissues in response to inflammation and are responsible for resolving existing inflammation and healing inflammatory damage, in addition to suppressing the initiation stages of inflammation. The effects are many and varied, including inhibiting chemotaxis, promoting clearance of neutrophils from tissues (diapedesis), promoting anti-inflammatory cytokine production like IL-10, promoting apoptosis and engulfment (efferocytosis) of macrophages, and other resolution pathways. Interestingly, one major response of the cholinergic anti-inflammatory reflex just discussed is to stimulate release of these mediators [161,162,163].

Although no studies have yet examined endogenous SPM levels in the bladder in either normal or inflamed conditions, we have shown the exogenous potential for these molecules to alleviate bladder inflammation and dysfunction in both BOO and cyclophosphamide-induced hemorrhagic cystitis [164,165]; the two bladder conditions discussed above which have been shown to lead to depression. Specifically, we have shown that all seven known SPM receptors are present in the bladder [164]. When administered during BOO, an Annexin-1A mimetic was able to block inflammation-inducing pathways, reduce bladder inflammation and normalize bladder function [165]. Excitingly, it also promoted faster and more complete functional recovery after surgical deobstruction, the experimental model of a transurethral resection of the prostate (TURP) and a clinically important example of lower urinary tract inflammation (albeit not linked to depression). In in vitro studies, we found Resolvin E1, Maresin R1 and Protectin D1 to promote epithelial wound/barrier repair in urothelia while in vivo they completely resolved bladder inflammation in response to cyclophosphamide. Finally, bladder function and fibrosis were examined in the cyclophosphamide model and Resolvin E1 restored these critical parameters back to control levels. Thus, several SPMs have been shown to be effective at resolving the very bladder inflammatory diseases known to cause depression, lending great optimism to the idea that these agents may be able to effectively and simultaneously relieve both the bladder and mood disorders.

One can increase the levels of the lipid SPMs in the body simply by ingesting EPA and DHA, or their precursors arachidonic acid and the much-touted omega-3 fatty acids. These precursors are widely available as cheap dietary supplements. Excitingly, researchers from the National institutes of Health in the USA recently found that supplementation of high levels of EPA or DHA improved depressive symptoms in patients with Major Depressive Disorder by an average of 64% and 71%, respectively [166]. Moreover, higher plasma metabolites levels were associated with less severe symptoms. SPMs and their precursors have little side effects and the main limitation of their use appears to be compliance by patients. This is likely because getting SPM levels high enough to be effective requires multiple large fish oil-filled gel capsules that must be swallowed several times a day. Excitingly, Thetis Pharmaceuticals Inc. has recently developed High Efficiency Amino Lipid Enabled Release (HEALER) technology able to transform these lipids into new molecules with vastly improved chemical and physical properties that greatly enhance their potential for pharmaceutical development. Dramatically reducing the size, number and frequency of doses to get an efficacious response holds great promise to normalizing SPM treatments for a variety of inflammatory-induced disorders including LUTS-induced anxiety and depression. Such treatments would also most likely alleviate the peripheral inflammation precipitating the depression in the first place, thus attacking LUTS-induced depression at multiple levels.

In considering treatments, there will undoubtedly be many inter-individual differences to take into consideration. Beyond comorbidities, gender is likely the most important to consider. While an in-depth discussion on sex differences in inflammation and depression is far beyond the scope of this review, it is worth noting that, in general, women experience higher levels of inflammation than men [167,168] while suffering disproportionately more from several psychiatric diseases [169], particularly mood disorders [170,171,172,173]. Future studies that examine sex differences in LUTS-induced mood disorders should prove to be quite revealing.

Finally, while we have mostly focused on the “out to in” mechanism by which peripheral inflammation in the urinary tract leads to neuroinflammatory induced mood disorders, it is essential to consider the bi-directionality of this relationship. This suggests that treating mood disorders may be an essential component to maximizing the treatment outcomes of patients seeing us for urological problems. Translation of this knowledge, which could involve teaming with mental health providers, is commonly done in pediatric voiding dysfunction clinics. So even if novel pharmacotherapies are not on the immediate horizon, translation into a clinical setting may be possible by taking full advantage of current resources in our treatment approach.

## 13. Conclusions

Although physicians have anecdotally linked LUTS, caused by a variety of diseases, to mood disorders such as depression for quite some time, only recently have large prospective studies confirmed this connection. As we have discussed, two major breakthroughs in different fields have led to an understanding of how this link is achieved. First, many LUTS-inducing conditions have an inflammatory component (UCICs) and, second, modern work on mood disorders has found that they are largely caused by neuroinflammation in the CNS, most notably in the hippocampus. Exciting new studies in at least two animal models of LUTS that have very well-established roles for inflammation, cyclophosphamide-induced hemorrhagic cystitis and bladder outlet obstruction, have demonstrated that these bladder conditions can directly cause hippocampal inflammation and depression, providing for the first time a mechanism explaining the link between LUTS and mood disorders. These new discoveries should lead to novel treatments and approaches for these diseases while interesting studies to come will undoubtedly determine the contribution of several possible pathways by which the inflammatory signal in the bladder is transferred into an inflammatory signal in the brain. Clearly, there is an immediate need to treat the psychiatric health of LUTS patients and it is likely that targeting the underlying inflammation, both in the bladder and in the brain, may bring both urological and psychological relief to these patients.

## Data Availability

Not applicable.
